# Covid-19 and the Brazilian Reality: The Role of Favelas in Combating the Pandemic

**DOI:** 10.3389/fsoc.2020.611990

**Published:** 2020-12-17

**Authors:** Luana Almeida de Carvalho Fernandes, Caíque Azael Ferreira da Silva, Cristiane Dameda, Pedro Paulo Gastalho de Bicalho

**Affiliations:** Psychology Graduate Program, Psychology Institute, Federal University of Rio de Janeiro, Rio de Janeiro, Brazil

**Keywords:** coronavirus, favela, community organization, territorial insurgencies, fundamental rights, Brazilian reality

## Abstract

The consequences of coronavirus in favelas in Rio de Janeiro (Brazil) point to social inequality as a structuring factor in Brazilian society. The contagion spread and multiple death cases reveal the multiplicity of existence ways that cohabit the urban context, indicating that in many of these scenarios, access to decent housing, drinking water, and minimum income is not a reality and recommendations from international health agencies are challenging to implement. Against government technopolitics that drive different forms of death to the poorest, black communities, and slum dwellers, territorial insurgencies indicate other paths for the construction of a dignified life and access to fundamental rights, targeted solidarity practices, territorial political organization and the construction of specific public policies to deal with the effects of the virus which takes into account the particularities and distinct realities of the territory. The experiences of community organization around Crisis Offices in the favelas, led by social organizations and supporting institutions, have guaranteed (i) food and personal hygiene items distribution, (ii) sanitization of alleys, (iii) dissemination of information on the virus, and (iv) political articulation for disputes in defense of life preservation in the favelas, in opposition of genocidal processes carried out by the state power. Such local spaces represent practices of resistance to the death policies undertaken by the state policies, which most are not configured as spaces for collective construction and disregard inequalities and different needs in these territories. That way, community associations are presented as an inflection point, a deviation from the normal course of modulated subjectivities by the social principles and practices of neoliberalism, with the indication that the most efficient way to deal with social crises is through the strengthening of the collective and the popular organizations.

## Introduction

The effects of the coronavirus pandemic in Brazil show the existence of a serious abyss, revealing that social inequality produces violations of rights and dictates who should live and who is destined to die and how their death is going to be. The pandemic process illustrates a death policy adapted by the State, called necropolitics by the Cameroonian intellectual (Mbembe, 2003), and points out some of the challenges for building a world where dignified life is not a privilege of a few, but a right of all.

This article is completed 9 months after the confirmation of community transmission in Rio de Janeiro, the second most populous city in Brazil—currently with more than 6 million inhabitants—and the first death due to the coronavirus pandemic. For us, Brazilians, the news about the outbreak of the disease caused by the new coronavirus, the Covid-19, begin to appear by the end of January 2020 in a massive way and soon after, in early March, the spread of the virus was an international health emergency by the World Health Organization (WHO) and therefore affirmed as a pandemic. The WHO suggested that the world should stop and isolate itself so that the process of contamination would slow down and not overload health systems, given the scarcity of resources to face it all over the world, especially in developing countries, marked by a history of colonization. It is worth mentioning that at the time of submission of the article, no vaccine had yet been developed to combat the virus and one million and 170 thousand deaths worldwide according to Pan American Health Organization^1^^,^^2^—with more than 157 thousand deaths in Brazil alone, a country that occupies the second place in the ranking of mortality by covid-19 despite underreporting, given the low testing of the population—one of the major problems in Brazil, warned by WHO even in the first months of the pandemic^3^.

The lack of energetic measures to combat the spread of the disease has enhanced a reality of crisis in Brazilian public health system—which has been living in recent years with overload and devaluation by the public power. Santos et al. (2020) point out that the early and cohesive closure of non-essential activities in Brazil has lasted little and the suspension of social distancing measures has been occurring asynchronously. Regarding the increase of contamination cases in Brazil, the study points out that these were the results of multiple factors, “including noncompliance, delayed implementation of social distancing measures, superspread through mass gathering events and the lack of coordinated control measures with neighboring municipalities” (p. 6). The study highlighted the lack of national coordination for the fight against Covid-19, and as the National Confederation of Municipalities (CNM)^4^ points out, the municipal non-pharmacological actions were prior to state and federal government guidelines, and the Ministry of Health published risk management strategies, risk assessment, guidelines and instruments to support decision-making in response to the Covid-19 pandemic at the local level only on May 11.

In recent years, especially after the impeachment of President Dilma Rousseff in 2016, there have been many setbacks in the field of social rights. The approval of Constitutional Amendment 95 in December 2016, which freezes investments in social areas, we have suffered from the intensification of the decay of our health systems, closure of beds and hospitals throughout the country. For many, the social collapse had already begun by analyzing this issue. The Brazilian Unified Health System is recognized worldwide for its capillarity, diversity of services, organization, especially for its public and free character. Political decisions have direct consequences on the maintenance and quality of services offered by SUS, making it even more difficult for the poorest population to access the right to health. Even in the cities where quarantine and social isolation were being decreed, many people could not interrupt their activities and so many others were forced to stop: given the continental and unequal reality of Brazil, where many work in informality to guarantee what to eat on a daily basis, how to adopt such restrictive measures, especially in view of a negligent government? As the Bolivian psychologist, Galindo (2020) affirms: in Latin America the coronavirus had exposed the colonial order of the world, highlighting that “Here the death sentence was written before the Covid arrived in a tourist plane” (p. 124). Even in the first months, the richest already said that the worst had passed^5^ even with the growing number of deaths among the poorest, who suffered from the difficulties to have access to any treatment. Costa et al. (2020) exemplify:

In Brazil, the supposed democratic character of the virus is questioned when one observes the data that the black and peripheral population has higher lethality rates than the rest of the population. In Rio de Janeiro, at the beginning of May, the data reveal that the lethality rate in the *Complexo de Favelas da Maré* is 30.8%, while in the *Leblon* neighborhood it reaches 2.4% (…). Nevertheless, (…) data (…) indicate that the number of hospitalizations and deaths of black and brown people has a higher rate of increase than that of white people (p. 2).

In a broader analysis, we see that in Brazil, the pandemic has never been about the richest; indeed, it is not about the poorest as well, but it does highlights the cruelty that our form of social reproduction of life imprints on society—for example, the first death by Covid-19 recorded in the country was a black woman, a domestic worker in a neighborhood of Rio de Janeiro's elite. In this sense, this article aims to problematize the relationship between the guarantee of rights and social inequalities in Rio de Janeiro's slums, from the scenarios that emerge with the new coronavirus pandemic, emphasizing that even in the face of government technopolitics that stimulates various forms of death to the poorest, blacks and slum dwellers, territorial insurgencies indicate other ways to build a dignified life and access to basic rights, guided by solidarity practices, territorial political organization and the construction of specific public policies to deal with the effects of the virus considering the particularities and different realities of the territory.

Our new invisible enemy “makes us see and speak” (Deleuze, 1986) about these inequalities. The big media started to publicize the solidarity actions and the government was strongly pressured to approve measures of attention to the favelas. In the House of Representatives, bills such as PL 1000/2020, which would institute the Covid-19 Attention Program in the Favelas; as well as the struggles for Emergency Aid, are signs of the dispute for the dignity of families in different spheres. Especially in the case of the city of Rio de Janeiro, a study dated September 9 revealed that if one compared the number of deaths per 100,000 inhabitants per Covid-19, if it were a country, the city would have the worst rate in the world (Brasil, 2020; Santos et al., 2020).

Invisible and exceptional the coronavirus was initially considered a “democratic virus”—an expression that composed many writings and TV news at the beginning of the spread. A widely spread disease that would reach everyone in an equal way, initially evidencing an evaporation of the nobility's security and, the fear of its contamination, overtook territorial and economic borders, with an idea of communion, of a possible world of greater solidarity where the virus would overcome the capital and the competitiveness entangled in it. “This virus is democratic and does not distinguish between poor and rich or between statesman and ordinary citizen” (Zizek, 2020, p. 25).

However, how can a virus be “democratic” (Zizek, 2020) in such an unequal country? The reality of Rio de Janeiro outskirts and slums shows itself to be another compared to noble areas; the orientations of epidemiologists, sanitarians and other scientific experts are incompatible with the material, financial and social structures that they possess, without basic conditions to follow food, isolation and hygiene prescriptions, not to mention that information about care often arrives biased and demoralizes the seriousness of the disease, treating it as a “little flu” (Löwy, 2020). Controlling contamination in so-called democratic countries could be a challenge, points out Sousa-Santos (2020), since each person is “free” to decide about their movement and other aspects of the operationalization of life. Just as, for Brazilians, access to information and public health services, for example, are also for everyone. A utopia, we know. “De-mo-cra-cy” four syllables and an elaborate phonetics, but that sometimes does not articulate and is inefficient to represent the right to equality and free and participatory exercise of life in the most different social classes (Bicalho, 2013).

It is important to point out that Brazil has one of the highest rates of social inequality (being in 10th position in comparison with other countries in the world, with an increase in inequality between the extremes of labor income distribution in 2019, according to the Institute of Applied Economic Research)^6^. To maintain the capitalist order, social Darwinism still permeates as an explanatory ideology to this phenomenon and massively affects the poor, blacks and working class, historically exploited by colonialism (Bolsanello, 1996). Those strongest, capable of adapting to the environment (and, here, surviving the pandemic) will survive—an addendum only to affirm that there is a government policy that dictates how some can live and others must die.

As Achille Mbembe points out, “My concern is with those forms of sovereignty whose central project is not the struggle for autonomy, but ‘the widespread instrumentalization of the human species and the destruction of bodies through terror in specific populations’ (Mbembe, 2003, p. 11). Politics that instrumentalizes us to better explain contemporary forms of subjugation of life to the power of death” (Mbembe, 2003, p. 36).

The specificities of the slums and peripheries are not something new. In the city of Rio de Janeiro, 763 favelas (IBGE, 2010)^7^ are officially accounted for, according to the Census conducted still in 2010, these have very different realities, but there are inequalities of access to basic rights as a common point, as there are places where the water supply is irregular and garbage collection is practically non-existent.

Slums, peripheries, communities in social vulnerability, places where poor people live, do not receive investments from the State on an equal basis like other regions of cities. Investments are lacking in essential areas, such as health, education, housing, lighting, among other services (Botelho et al., 2020, p. 4).

As described in the online newspaper *Maré de Notícias*^8^ —one of the media vehicles for the events and daily reality of the *Complexo de Favelas da Maré* in Rio de Janeiro, where more than 140,000 people live—it highlights that in the slums most houses are small with few rooms and many people, without air circulation, making it difficult to respect the prevention measures proposed by national and international health agencies.

The initial orientation to social distancing already warned about the risk of social upheaval in the slums, with the perspective of marked impoverishment: seven out of 10 families would have their incomes compromised in a first analysis^9^. There are many reasons for this, including precarious, informal, autonomous, outsourced labor relations or services provision through platforms. Such precariousness, as Assunção-Matos and Bicalho (2016) point out, is a process that articulates itself with poverty.

As Forrester (1996) states:

We live in the midst of a masterly decoy, a world that has disappeared that we persist in not knowing as such and that certain artificial policies intend to perpetuate. Millions of destinies are destroyed, annihilated by this anachronism caused by reluctant stratagems, destined to present our most sacred taboo as imperishable: work (…) we participate in a new era without being able to observe it. Without admitting and not even realizing that the previous era has disappeared (p. 7)

In this sense, small businesses in these territories have been quite affected by the effects of the pandemic, influencing the reduction of work and of family income itself, considering that 46% live off the income of their own business; and 15% have opened a new business in the last 12 months, mostly out of necessity. Due to a high number of informal establishments, accessing credit lines is more difficult. In fact, there is an entire service based economy that supports many of the families in these locations: manicures, bricklayers, bakers, housekeepers. Immediately, an obstacle to the guidelines of quarantine and social isolation, since “the bread of each day” depends on daily work. All this reality, exposed by the coronavirus, has always existed in these places. As Dornelles (2017) points out:

What happens, especially in times of barbaric capitalism, as adopted by the neoliberal order, is that the human contingents that are in a situation of vulnerability, of social exclusion, are increasing all over the world. They make up a multitude of human beings who are now identified as enemies of order and dangerous, whose existence and living conditions are not treated as a result of this model of capital accumulation, but rather as segments to be criminalized and punished (p. 123).

The majority of favelas arise from an unequal reality that imposes itself on workers in a brutal way, limiting their access to fundamental rights such as the right to housing (Valle et al., 2015). And there have been many attempts in the history of the slums where the residents have held the public power responsible for improvements, but there have also been many times when the public power has acted tirelessly in the destruction of slums, in the demolition of houses, and in the rupture of democratic dialogues with the population. The understanding of the slum as a problem to be extinguished or at least controlled in the context of its population growth has been present in public authorities since the beginning of the 20th century (Melicio et al., 2012).

Thus, the logic of “we for us” is reigning in many of these territories, not as a dead word, but as a daily practice of invention of a world where life is possible to be lived—a philosophy of existence that is guided by sharing, by the strengthening of the collective, which enforces the principles of “ubuntu,” an African philosophy that points to an ethos of solidarity and interdependence with others. When the cobwebs unite, says an Ethiopian proverb, they can tie a lion.

Ubuntu situates individuals within a web of relationships that is born of identifying with others and acting in solidarity. It is by sharing a way of life with others that individuals' come into existence'. We exist because our social connections remain strong, extending beyond family to embrace our clan, village and entire community (Seifu Estifanos et al., 2020, p. 1).

The new coronavirus not only causes sanitary and economic changes, but it also makes visible what has been put as a priority and challenges humanity in the construction of a new reality; in this sense the pandemic can be considered as an event, in the Foucaultian vision, and the threat of contagion by SARS-CoV-2 as a powerful device. Foucault (2008) affirms the event as that which when it erupts, causes discontinuities in the field of knowledge-power, turning certain discourse possible by changing the epistemic of an time. And as for devices, they are machines, networks, always partial, momentary (never universal and eternal) that respond to certain effects as they are in a continuous process of object production (Barros et al., 2009). A device, therefore, that questions the right to life, the right to dignity (Universal Declaration of Human Rights, 1948), and questions the function of the State, our empathic capacity, and our processes of choice in the face of the death threat. The coronavirus gives visibility to the different realities marked by social inequality, as in the case of Brazil. As the Portuguese philosopher Gil (2020) points out, the pandemic is not about the fear of death, but above all, the fear of absurd death.

## #Covid19Nasfavelas

The city of Rio de Janeiro has the largest number of people living in slums in the country, representing 22.03% of the population. Comparing the 2010 Census with the 2000 Census, there was a 27.5% growth. Since the beginning of the pandemic in the city of Rio de Janeiro, some legislative initiatives have been proposed by the federal, state and municipal governments to contain contamination by the virus in the slums, many of them created in dialogue with social movements and universities, with the aim of creating emergency attention plans for the slums, in an understanding of the importance of the State taking responsibility for ensuring a minimum for a quarantine with dignity. This includes broad issues from access to water, food and minimum income, as well as the safety of residents, with a Law Project banning police operations in the slums while the pandemic remains. It is important to point out on this point that during the month of May, one of the most serious moments of the pandemic, police operations killed dozens of young black people. In *Complexo do Alemão*—a neighborhood that houses one of the largest groups of slums in the municipality, with about 120,000 residents—in a single operation 12 people were killed by the police, for example. Although important initiatives have been filed in the legislative houses, most of them have not been approved and those that were approved—late—are still not being implemented. The slowness of implementation of the measures makes explicit the relationship that governments establish with such territories, where efforts to preserve life are less frequent than the imminence of death.

In the absence of effective actions by governments to control the pandemic and with the progressive increase of cases and deaths, another sector of the slums has also presented measures to “combat” the coronavirus. Operators of the retail drug trade in several places have made strong recommendations for social isolation, prohibiting the occupation of public roads within slums and agglomerations^10^. Acari, another neighborhood located in the city of Rio de Janeiro, people supposedly linked to drug retailers would be using a sound car, called *Carro da Lapada*, to warn residents that it is not vacation time, but protection, including setting parameters for curfews and indicating who is allowed to go out on the streets. This car would also be monitoring crowds and making threats—the *lapadas*—a local slang to connote the use of physical violence in case of non-compliance with the recommendations, since “if you do not embrace the talk, the talk embraces you”^11^.

Understanding the reticular and multidimensional reality helps us to advance in a more propositional posture on the reality of the slums, which are heterogeneous territories where many realities and social markers cohabit. It is also important to understand that problematizing the reality of the slums does not mean addressing only issues related to precariousness and poverty; it is worth noting that the residents of Brazilian slums have a consumption power of 119.8 billion reais per year (equivalent to 21 billion dollars), a mass of income that surpasses countries such as Uruguay, according to the survey “Economy of the Slums” conducted by the *Instituto Locomotiva* and *DataFavela*^12^. Faced with this scenario, the strength of the favela in terms of power of resistance, community articulation, solidarity and social engagement should be highlighted.

In the context of a cultural capitalism that expropriates and resells ways of life, wouldn't there be a growing tendency, on the part of the so-called excluded, to use life itself, in its precarious subsistence, as a vector of self-worth? (…) Their only capital being their life, in their extreme state of survival and resistance, is that which they made a vector of existentialization, is that life that they capitalized and thus self-valued and produced value (Pelbart, 2003, p. 22).

Following the course of actions taken in relation to the coronavirus, the articulations made in these territories are notorious, to the point of recognition on March 31 by one of the Health ministers of the period, Luiz Henrique Mandetta, at a press conference:

Congratulations to the communities of Rio de Janeiro. Congratulations to the favelas, the communities, and I know them. I studied there. I studied there. I did voluntary action both there in *Vidigal* and in *Rocinha* when I was a medical student. The other day I went to launch the sexually transmitted diseases program there in Rocinha with young people from the community. Congratulations *Maré*, congratulations for the work you are doing and the example of dignity, behavior, intelligence. From the wisdom class you are giving. In the dynamic, *Heliópolis* in São Paulo, all of them. *Paraisópolis*. All of them. I'm talking about Rio de Janeiro because I stayed 10 years in that city^13^.

Mandetta's speech is controversial because it is not part of a context where the state operates policies to guarantee rights for the population, but it is aligned with neoliberalism because it bets on a rationality where the state does not take responsibility as a sponsor of social welfare and care for the population. It is important to point out that collectives and leaders strongly reprimanded the then minister and the policy operated in the period.

In March, part of the recommendations listed by the Ministry of Health, led by the aforementioned minister, and by Brazilian leaders in the face of the pandemic that was approaching our country, did not include the favelas. The first attempt to organize the demands of these territories came, not by chance, from the coalition between leaders of the *Complexo do Alemão, Cidade de Deus, Complexo de Favelas da Maré, Rocinha* and *Santa Marta*, with researchers from the universities Federal University of Rio de Janeiro, Pontifical Catholic University of Rio de Janeiro and State University of Rio de Janeiro, in dialogue with the public health research institution Oswaldo Cruz Institute^14^. The plan gathers initiatives in the preventive dimension, indicates the need for protocols for medical care, points out parameters for the coordination of territorial actions and defends the construction of an Office of Crisis of Attention to the Favelas. Even though, when the plan was presented and delivered to the public power, specific actions were already being taken by it, the articulation of a plan that thought about the slums in their amplitude and diversity and proposed organizational and political ways to face the pandemic only happened in the meeting of researchers (mostly from public institutions) with territorial leaders. The role of universities and research institutes at this time is to reaffirm their social function and strengthen policies that are in line with the interests of Brazilian society, without losing sight of the need to treat unequally those who are unequal to the extent of their need; that is, to pay attention to the principles of isonomy for access to rights, assured by the Brazilian Federal Constitution of 1988.

One of the main measures proposed in the above plan is the articulation of a social support network. Based on the understanding that the pandemic brings impoverishment to many families, it becomes essential to defend solidarity measures, such as the distribution of basic food baskets, drinking water, masks and gloves for individual protection, hygiene and related materials, but also the fight for public policies to access income, such as the release of the emergency aid. Still in April, the House of Representatives voted and approved the release of the benefit, whose values vary between 600 and 1,200 reais (equivalent to between 112 dollars and 224 dollars). The beginning of the payment of the first installment of the emergency aid took place on April 9, among the bureaucracies established for the access to the benefit were the access to the internet, through website and application and the use of bank account—two difficult points considering the Brazilian reality. The result was the formation of immense queues and agglomerations at *Caixa Econômica Federal* and *Lotéricas* branches, which caused many families to spend weeks trying to receive their benefit, without success. Recently the Federal Government informed the endure of the benefit payment until the end of 2020, but in the amount of 300 reais (equivalent to 56 dollars), which is insufficient for the purchase of food from the basic basket, for example.

The social inequalities discussed here have intimate relations with historical and contemporary political processes, which have existed since long before the pandemic and will still exist after its end. Thus, it is possible that the experience of the pandemic in Brazil potentiates an unprecedented crisis. The Ministry of the Economy announced in an optimistic perspective, a drop of 4.7% in Gross Domestic Product (GDP)^15^. Financial institutions such as JP Morgan and BTG Pactual, project a drop of 7% in Brazilian GDP^16^^,^^17^. It is evident that considering the current expectation of the Federal Government, the country will suffer the biggest fall in GDP in history, since similar percentages occurred only in 1981, with a decrease of 4.39% in the value of GDP, which indicates a deterioration in the quality of life of a specific population.

It is not by chance that in Brazil, different from countries like Italy, Portugal, England and France, the concentration of cases of lethality by coronavirus is not marked by differences in age group. Here, what determines who lives or dies due to the complications of the virus, are socioeconomic factors with a very strong racial component among the “risk determinants.” Goes et al. (2020) indicate that, while on the one hand the absence of concrete data collected by the State makes it difficult to map the real number of black people who are contaminated, the history of low access to health and greater vulnerability to chronic diseases points to concerns for the black population to prevent and combat covid-19. There are a number of factors that prevent access to the correct diagnosis and appropriate treatment and it is no coincidence that the first death in Rio de Janeiro was of a domestic employee, contaminated by the owner of the house where she worked, who was in Europe shortly before the pandemic^18^. The data reveal, therefore, that the neighborhoods with more blacks concentrate more deaths than the neighborhoods with fewer blacks, in absolute majority^19^. Even so, the Ministry of Health replies that there is no information on how many cases were confirmed by race/color, nor the number of tests from racial groups. In Rio de Janeiro, a lawsuit was filed to determine that the race/color markers of infected and dead were recorded, so that more concise data can be produced on vulnerable groups to the pandemic. Federal Judge Dimitri Wanderley's decision holds the Union responsible for issuing guidelines for the mandatory completion of data throughout the country^20^. The decision has not yet had any effect on the results that are exposed. Without general guidance and supervision, we continue to operate in the logic of concealment of data to build alternative and false realities, where markers of race and class do not influence in cases of death and contagion, producing the false idea of a virus that is democratic. This repeats itself in several other regions of favelas or peripheries, not only in Rio de Janeiro.

It is important to point out, at this time, that underreporting is robust, and can be even greater in the case of slums and peripheries, since, besides overcrowding in the public health network, access to testing is still very expensive in the private sphere. Without strengthening the Unified Health System and the consequent expansion of tests and increase of vacancies in public Treatment Centers, it is clear that the most affected population will be the poorest.

At the same time that official data are disseminated, residents have also conducted their own research on infected and dead people^21^, such as measures to fight fake news. In May, for example, a community media outlet, the newspaper *Voz das Comunidades*, of the *Complexo de favelas do Alemão*, launched an application with information about the new coronavirus in order to provide real-time access to reliable information and news verification. The application was made with funding from the American Consulate in Rio de Janeiro^22^. Regarding the reliability of the information regarding the contagions that occurred in the *Rocinha* slums (100,000 inhabitants) and in the *Complexo de Favelas da Maré* (140,000 inhabitants), there are studies that point out that the number of deaths can be up to three times greater than what was disclosed by official agencies. The deaths in these territories already exceed the numbers of many cities in the metropolitan region of Rio de Janeiro, such as Niterói (with an estimated population of 515,000 people) or São Gonçalo (with an estimated population of 1,100,000 people).

In the midst of the crisis caused by the new Coronavirus, people living in slums and peripheries have been mobilized through community articulation and strengthening of solidarity networks to fight the virus and reduce social and economic impacts in the territories. The pandemic has demanded actions to combat harm and mitigate it in different spheres, while the issue of treatment to COVID 19 is mainly managed by the SUS, actions related to the dissemination of reliable information, prevention and social assistance, for example, have been enhanced by the articulation among various civil society actors such as universities, NGOs and private companies. The initiatives developed are several and enclose the fight against fake news, as previously discussed, enhancement of preventive actions with local public health institutions, collection of donations, sanitization, registration of low-income families, production of knowledge, and shelter to residents.

In early April, residents of the *Santa Marta* slum sanitized the streets, from articulation with the local business, as one resident says: “I talked to some friends of mine, they paid for the equipment and I bought glove, simple materials, and got some donations for chemical materials, which will end. Our slum is the first in Brazil sanitized with the same equipment as China, by the residents”^23^, and several slums made contact to learn how to use the equipment. Other actors like the Public Defender's Office of Rio de Janeiro and the State Public Prosecutor's Office also carried out important actions: almost 2 months after the beginning of the pandemic, the entities got an injunction in court that forces the public power to regularize the water supply in the slums. Small victories like this one walk toward a horizon where the favela can live with dignity.

Still considering the localized actions, the food collection campaigns have been mobilized by several institutions, such as the *Central Única das Favelas* (CUFA)^24^ and the Marielle Franco Institute^25^; the latter has launched a map to give visibility to initiatives to combat the coronavirus in the slums and peripheries of Brazil. The mobilizations also took place through musical live shows in partnership with artists and private companies. Another important action to be mentioned is the *Mães de Favela* (Mothers of the Slums) campaign held at the beginning of April by CUFA, built from research conducted by the *Instituto Locomotiva* and *Data Favela*, which highlights that Brazil's slums have 5.2 million mothers, in which it was projected that more than 70% would be without income during social isolation. The research published in BBC News Brazil highlights the place of vulnerability and the social role of women living in slums in supporting and caring for their children and elderly, from an intersectional perspective:

The most fragile in society are the slum dwellers. The most fragile among slum dwellers are women. And the most fragile among women are mothers. Why? Because they take care of their children, often working in informal jobs, sewing, nailing, and still take care of the old. Because all the old people—90% of the old people in the slums—are cared for by women: whether they are daughters-in-law or daughters^26^.

This situation is a concrete example of what expresses the concept of necropolitics, formulated by the Cameroonian philosopher Mbembe (2003). In Rio de Janeiro, the expression has already been explored by those who discuss public security policy, the war on drugs, the extermination of black youth, and so many other issues that are related to the way the state deals with the favela and periphery territories. Applied to the moment of the pandemic, we can place the concept on another analytical level, expanding the understanding of a life's government that is present in these places by exterminating lives in the most conventional ways (by police operations or mass imprisonment) but also from a historical construction marked by absences, lack of responsibility of the State, barriers to development, lack of investment and specific planning and hiding data about the reality of a people.

Therefore, the understanding of a policy of death that is actively operated gains a special contour, while dignified conditions for the exercise of care, which often contradict the orientations of health agencies and specialists, are not guaranteed. Nevertheless, it also presents itself in a more cunning dimension, in the exercise of unaccountability over the lives of the most vulnerable, in the disinvestment that has been made in recent years in social protection measures, in the deregulation of the labor world, and in the freezing of investment in social areas such as science and technology and public health, one of the hardest effects of neoliberal tentacles on state governance.

Faced with the uncertainties of the scenario, the networks of solidarity have played the role that the State is not playing when it comes to facing the spread of the new disease. From the donation, collection and distribution of food and hygiene products, to the manufacture of masks, and the dissemination of important sanitary information regarding contagion, the community force has been the great mechanism to minimize the damage of this pandemic conjuncture and increase collective resistance and the feeling of belonging. To this end, these networks in the slums have been leading actions in the fight against Covid-19, making a difference to overcome one of the largest public health crises in the world.

## Discussion

A crisis contained within other existing ones—historically—and once again, socioeconomic factors, closely linked to the color-race class, determine who lives and who dies, not only in Rio de Janeiro, despite this being part of the scenario. We point out that the underreporting of Covid-19 has produced distorted realities, and such markers have not been taken into account by statistics and have become operators of death policies. The same would be to say that, by not recognizing the specificities, as it is lacking—it does not plan improvements, does not invest, does not guarantee decent conditions of care, freezes investments in social and health areas, and deregulates labor rights—the State actively operates to exterminate the poorest people and increase social inequality.

The pandemic has been an important device to make the harsh reality of inequality of the slums and peripheries in our country be seen and talked about. But does it put these favelas on the map once again? The “rediscovery” of the slums that has been happening in the mainstream media and in government actions does not happen exactly because of the concern with the slums and their inhabitants themselves. At the beginning of the pandemic, some people said that the big problem in Brazil would be the slums, in purely racist, eugenic comments in a logic that imagined that the slums would contaminate the rest of the country. This “rediscovery,” therefore, has given way to a concern that is more intense with those who live outside these spaces than with those who live there, as if the slum dwellers and peripherals were the great danger for the expansion of the coronavirus.

Do these analyses consider that the traces of the first death show that the path is often the opposite? Or that the data indicate that, although “those who pay the bill” are the poorest, black, slum residents and peripherals, the blame for the contagion lies not with these people, but with the government which resists dealing with their responsibility and have kidnapped our country for private interests of a few (Löwy, 2020)? The slums, on the other hand, are the “social sector” that is most organized and active in the fight against the coronavirus. An analysis of the mapping of the Marielle Franco Dictionary of Slums^27^ reveals dozens of territories organized into territorial actions. At the same time, the initiative to think about public policies to confront the chaos that is being placed has also been of the favelas, articulated in their collectives and leaders. In several favelas in Rio de Janeiro, like *Chapéu Mangueira* and *Babilônia*, there are initiatives for psychological assistance to residents, organized by community leaders in dialogue with volunteers, for example^28^.

As the virus demands patience from us—since the uncertainties related to its therapy are still great—it reveals even more a sense of urgency because “those who are hungry are in a hurry.” This sentence exemplifies the inequalities stamped on Brazilian society and the need for a paradigmatic change in the sphere of public management. The coronavirus shows the particularities of the slums and peripheries and the government's negligence in the face of the demands of the poorest. Exclusions, precarious housing, rents compromised by interrupted work, and, as observed, these are not only measures of isolation of these people, but also the suspension of services to provide that prevent them from working. Poverty is spreading.

One concern is shared by residents and activists alike, as well as by public authorities: impoverishment and a decline in the quality of life of slum dwellers is a major problem. The greatest divergence is about the exits to be presented: if, on the one hand, the adoption of awareness campaigns, the lockdown and maintenance of emergency aid is advocated, on the other hand there are signs of exits that may cause even more violations of rights. The most extreme example of this was the attempt to militarize the social issue undertaken by the mayor of Rio de Janeiro, who requested a federal action to close down businesses in the favelas, even pointing out that he could hardly deal with the place without the mediation of the Military Police and its weapons^29^. The demand for the presence of the public power here does not require more truculence, but the construction of mechanisms that guarantee at the same time the full protection of families in these territories and their right to quarantine with dignity (with distribution of food items, hygiene, sanitization of public roads, release of Internet networks, release of emergency aid) and the construction of plans for access to health services for all who demand, with testing, orientation, hospitalization and care for all. A healthy life has been incompatible with the capitalist structure put in place. Under quarantine conditions, individuals living in slums and peripheries suffer and more than ever, and they point out that equality does not exist, not even by the law.

Despite so much neglect and violation of rights, slum dwellers recognize their needs and even more their strength; they mobilize their human capital, activate and strengthen their networks, and develop preventive and emergency actions, as well as request and, in their own way, summon important actors to hold public power accountable for guaranteeing the rights to life. The important role of community association, whether in donations or in proposing public policies, is evident. It is a turning point, a deviation in the normal course of subjectivities modulated by the principles of neoliberalism, with the indication that the best bets of ways out of the crisis are organized in the meeting with the collective. Boaventura Sousa-Santos^30^ points out that the reinvention of democracy involves an expansion of democracy in this community dimension, in neighborhoods, in civic education oriented to solidarity and cooperation, in the fight against the extremely strong ideals of entrepreneurship and competitiveness that prevail today. The exits found by the suffering population are the actions of solidarity, of awareness, of specific claim, which are extremely important, however, only the State is the guarantor of rights through the creation of localized public policies.

## Author Contributions

All authors listed have made a substantial, direct and intellectual contribution to the work, and approved it for publication.

## Conflict of Interest

The authors declare that the research was conducted in the absence of any commercial or financial relationships that could be construed as a potential conflict of interest.

## Footnotes

^1^https://www.paho.org/pt/covid19 (accessed October 30, 2020).

^2^https://bityli.com/btg4K (accessed October 30, 2020).

^3^https://bityli.com/rqlyS (accessed October 30, 2020).

^4^https://www.cnm.org.br/comunicacao/noticias/pesquisa-da-cnm-com-mais-de-4-mil-municipios-mostra-resposta-do-poder-publico-local-a-covid-19 (accessed September 11, 2020).

^5^https://bit.ly/3dVWUre (accessed May 13, 2020).

^6^https://static.poder360.com.br/2019/12/mercado-de-trabalho-20193T-ipea.pdf (accessed May 4, 2020).

^7^https://cidades.ibge.gov.br/brasil/rj/rio-de-janeiro/pesquisa/23/25359

^8^https://mareonline.com.br/mare-de-noticias/sem-espaco-para-isolamento/ (accessed April 30, 2020).

^9^https://exame.com/economia/renda-cai-para-7-em-cada-10-familias-nas-favelas/ (accessed March 31, 2020).

^10^https://bit.ly/2TiEfOk (accessed April 28, 2020).

^11^https://odia.ig.com.br/rio-de-janeiro/2020/04/5904264-carro-da-lapada–criminosos-anunciam-toque-de-recolher-em-acari.html#foto=1 (accessed April 29, 2020).

^12^https://glo.bo/36fg9t5 (accessed January 31, 2020).

^13^https://bit.ly/3g5ug98 (accessed March 31, 2020).

^14^https://ufrj.br/noticia/2020/05/05/ufrj-participa-de-plano-de-acoes-contra-covid-19-nas-favelas (accessed May 27, 2020).

^15^https://oglobo.globo.com/economia/com-pandemia-governo-preve-queda-de-47-no-pib-em-2020-24424322 (accessed May 14, 2020).

^16^https://glo.bo/2LFNJis (accessed May 11, 2020).

^17^https://bit.ly/3bGt6xm (accessed May 16, 2020).

^18^https://glo.bo/3cKTyab (accessed March 20, 2020).

^19^https://bit.ly/2X33t4s (accessed May 7, 2020).

^20^https://bit.ly/2ADFCRf (accessed May 7, 2020).

^21^https://bit.ly/2WHESmR (accessed May 11, 2020).

^22^https://bit.ly/2WGNdqH (accessed May 11, 2020).

^23^https://glo.bo/3g1wCFN (accessed April 5, 2020).

^24^https://www.cufa.org.br/sobre.php (accessed April 5, 2020).

^25^https://www.institutomariellefranco.org/ (accessed April 5, 2020).

^26^https://www.bbc.com/portuguese/brasil-52131989 (accessed April 5, 2020).

^27^https://bit.ly/2z8ZWcT (accessed September 12, 2020).

^28^https://glo.bo/3dWwPIv (accessed September 12, 2020).

^29^https://glo.bo/3g2si9m (accessed September 12, 2020).

^30^https://www.brasil247.com/blog/virus-tudo-o-que-e-solido-se-desfaz-no-ar (accessed April 5, 2020).
